# Interventions to Promote the Quality of Life and Psychological Well-being in Chronic and Developmental Psychopathologies

**DOI:** 10.3389/fpsyg.2021.640175

**Published:** 2021-02-12

**Authors:** Luana Sorrenti, Pina Filippello

**Affiliations:** Department of Clinical and Experimental Medicine, University of Messina, Messina, Italy

**Keywords:** quality of life, psychological well-being, developmental psychopathologies, interventions, prevention

Many studies have highlighted the role that the quality of life (QoL) plays for the individual's mental and physical well-being, preventing the onset of psychopathologies and chronic diseases (Marchini et al., 2018; Conversano, 2019; Martino et al., 2019, 2020a,b,c; Yoo and Ryff, 2019), which certainly have a serious impact on the general functioning of each individual. In fact, chronic diseases are frequently associated with psychopathologies, as they affect the cognitive, emotional and relational functioning, in all life contexts (Ingram and South, 2020; Lenzo et al., 2020; Marchini et al., 2020; Martino et al., 2020b; Vicario et al., 2020; Wang et al., 2020), also regardless the age.

This concept challenges psychologists to collaborate with other health professionals in order to structure interventions aimed at ensuring the better quality of life for the individual with different disabling diseases, since the early stages of the life cycle. In this regard, studies concerning children and adolescents with neurodevelopmental disorders highlight how it is necessary to promote learning, inclusion and consequently well-being in school and family environment. For instance, missing the adequate psychological support, children with oppositional defiant disorder (ODD) and attention deficit hyperactivity disorder (ADHD) can develop emotional disturbances, conduct disorders, and antisocial personality disorder in adulthood (Chen et al., 2020; Di Giuseppe et al., 2020). These psychopathological conditions are frequently associated with management difficulties of parents, who, in turn, risk to develop psychological symptoms and psychopathologies, such as anxiety and depression (Manti et al., 2019). Moreover, further difficulties in managing occur in school context, due to dysfunctional children behavior (Albertova, 2020; Han et al., 2020). Therefore, it is needed to structure parent and teacher training aimed at managing the disadaptive patterns, as well as reducing the oppositional, hyperactive and inattentive behaviors which characterize these specific disorders.

Psychological signs and symptoms are also associated to Specific Learning Disorder (SLD), negatively affecting their functioning and academic and social adaptation. Scientific evidences highlighted children with SLD have internalization problems, as anxiety and depression and externalization problems, as anger and aggressive behavior (Ryan, 2006; Mogentale and Chiesa, 2009; Ghisi et al., 2016; Mammarella et al., 2016; Sorrenti et al., 2019). If timely interventions are missing, internalization and externalization issues can evolve over time, eventually leading to psychosocial maladjustment. Instead, it is known low self-esteem, academic failure, school phobia, school refusal, school dropout, and absenteeism may be found among students with SLD (Venkatesan, 2017; Filippello et al., 2020b). These studies underline the importance of structured early interventions aimed at developing and enhancing learning strategies, such as metacognitive ones (Zumbrunn and Bruning, 2013; Filippello et al., 2016b), useful to promote learning and scholastic well-being. In fact, school is one of the main contexts in which it is necessary to promote the psychological well-being of all students, with particular regard to those with special educational needs (SEN). Indeed, they are more vulnerable, at risk of school failure and they could develop psychological illnesses in comparison with typically developing peers.

Unfortunately, many students have internalizing problems that are not adequately addressed with psycho-educational interventions with the consequence that, getting worse, these problems can evolve into more serious and disabling psychopathologies, such as depression. Many researches highlighted several individual variables (e.g., dispositional optimism, personality, positive or negative affectivity, adaptive or dysfunctional explanatory style, emotional intelligence, frustration tolerance, adaptive, or maladaptive perfectionism) which play an important role in academic success, influencing the student's sense of self-efficacy, academic engagement, and psychological well-being (Saklofske et al., 2012; Filippello et al., 2016a, 2018a,b; Steinmayr et al., 2016; Damian et al., 2017). These studies also showed that if a negative circle exist between dysfunctional individual variables (e.g., pessimistic explanatory style, frustration intolerance, or maladaptive perfectionism) and school failure, the student can experience a sense of helplessness and, in the most serious cases, depression. Furthermore, these dispositional variables are often associated with contextual variables, such as relationships with parents and teachers. If these relationships are positive and supportive of the students' needs, there play a protective role against the development of psychopathologies; if not, being negative, they hinder the needs of adolescents, predicting psychological illness, school and social maladjustment, persisting over time (Wang, 2012; Haerens et al., 2015; Diaferia et al., 2018; Filippello et al., 2019a,b, 2020a,b; Buzzai et al., 2020). Finally, due to the chronic disorder and the severity of symptoms, psychological interventions could not be enough, and pharmacological treatments should be performed to promote QoL and psychological well-being.

In light of the above, effective interdisciplinary approach and collaboration between Psychology and Medicine are necessary, in order to better cure individuals suffering from chronic diseases and psychopathological disorders, in different contexts and lifelong, from childhood and adolescence. Thus, it is needed to timely identify specific psychological signs and symptoms, at early stages of life. A preventive perspective could significantly promote QoL and well-being with specific regards to developmental psychopathologies.

## Author Contributions

LS conceived the idea and made a significant contribution by drafting the manuscript. PF critically revised the manuscript and approved the final version to be published. All authors contributed to the article and approved the submitted version.

## Conflict of Interest

The authors declare that the research was conducted in the absence of any commercial or financial relationships that could be construed as a potential conflict of interest.

## References

[B1] Albertova S. M. (2020). Emotional Dysregulation as an Aspect of ADHD: How to support the well-being of students with ADHD in schools. Pedagogika Przedszkolna i Wczesnoszkolna 81, 87–95.

[B2] Buzzai C. Sorrenti L. Orecchio S. Marino D. Filippello P. (2020). The relationship between contextual and dispositional variables and well-being and hopelessness in school context. Front. Psychol. 11:533815. 10.3389/fpsyg.2020.533815 33013591 PMC7516339

[B3] Chen Y. L. Ho H. Y. Hsiao R. C. Lu W. H. Yen C. F. (2020). Correlations between quality of life, school bullying, and suicide in adolescents with attention-deficit hyperactivity disorder. Int. J. Env. Res. Pub. Health 17, 32–62. 10.3390/ijerph17093262 32392842 PMC7246627

[B4] Conversano C. (2019). Common psychological factors in chronic diseases. Front. Psychol. 10:2727. 10.3389/fpsyg.2019.02727 31866912 PMC6909152

[B5] Damian L. E. Stoeber J. Negru-Subtirica O. Băban A. (2017). Perfectionism and school engagement: a three-wave longitudinal study. Pers. Individ. Dif. 105, 179–184. 10.1016/j.paid.2016.09.044

[B6] Di Giuseppe M. Prout T. A. Rice T. Hoffman L. (2020). Regulation-focused psychotherapy for children (RFP-C): advances in the treatment of ADHD and ODD in childhood and adolescence. Front. Psychol. 11:572917. 10.3389/fpsyg.2020.572917 33224067 PMC7669541

[B7] Diaferia C. Artoni V. Rollo D. (2018). “Scaffolding in teacher-student interaction: an operationalization for evaluation in educational and clinical settings,” in IEEE International Symposium on Medical Measurements and Applications (MeMeA) (Rome), 1–5. 10.1109/MeMeA.2018.8438694

[B8] Filippello P. Buzzai C. Costa S. Orecchio S. Sorrenti L. (2020a). Teaching style and academic achievement: the mediating role of Learned Helplessness and Mastery Orientation. Psychol. Sch. 57, 5–16. 10.1002/pits.22315 33136418

[B9] Filippello P. Buzzai C. Costa S. Sorrenti L. (2019a). School refusal and absenteeism: perception of teacher behaviors, psychological basic needs, and academic achievement. Front. Psychol. 10:1471. 10.3389/fpsyg.2019.01471 31316431 PMC6610479

[B10] Filippello P. Buzzai C. Messina G. Mafodda A. V. Sorrenti L. (2020b). School refusal in students with low academic performances and Specific Learning Disorder. The role of self-esteem and perceived parental psychological control. Int. J. Disability Dev. Educ. 67, 592–607. 10.1080/1034912X.2019.1626006

[B11] Filippello P. Buzzai C. Sorrenti L. Costa S. Abramo A. Wang K. T. (2019b). The Italian version of the family almost perfect scale: psychometric characteristics and relationships with academic engagement, self-esteem, and personal perfectionism. Appl. Dev. Sci. 10.1080/10888691.2019.1647106. [Epub ahead of print].

[B12] Filippello P. Harrington N. Costa S. Buzzai C. Sorrenti L. (2018a). Perceived psychological control and school learned helplessness: the role of frustration intolerance as a mediator factor. Sch. Psychol. Int. 39, 360–377. 10.1177/0143034318775140

[B13] Filippello P. Sorrenti L. Buzzai C. Costa S. (2016a). L'Almost Perfect Scale-Revised: un contributo all'adattamento italiano. Giornale Italiano di Psicologia 4, 911–930. 10.1421/85584

[B14] Filippello P. Sorrenti L. Buzzai C. Costa S. (2018b). Predicting risk of school refusal: examining the incremental role of trait EI beyond personality and emotion regulation. Psihologija 51, 51–67. 10.2298/PSI170526013F

[B15] Filippello P. Spadaro L. Sorrenti L. Mafodda A. V. Drammis L. (2016b). Processi metacognitivi e di pianificazione in bambini con disortografia [Metacognitive Processes and Planning in Children with Dysorthography]. Psicologia Clinica dello Sviluppo 1, 83–102. 10.1449/83131

[B16] Ghisi M. Bottesi G. Re A. M. Cerea S. Mammarella I. C. (2016). Socioemotional features and resilience in Italian University Students with and without Dyslexia. Front. Psychol. 7:478. 10.3389/fpsyg.2016.00478 27065220 PMC4814487

[B17] Haerens L. Aelterman N. Vansteenkiste M. Soenens B. Van Petegem S. (2015). Do perceived autonomy-supportive and controlling teaching relate to physical education students' motivational experiences through unique pathways? Distinguishing between the bright and dark side of motivation. Psychol. Sport Exerc. 16, 26–36. 10.1016/j.psychsport.2014.08.013

[B18] Han G. T. Chen Y. L. Tsai F. J. Gau S. S. F. (2020). Temporal and reciprocal relations between ADHD symptoms and emotional problems in school-age children. J. Atten. Disord. 24, 1032–1044. 10.1177/1087054718787891 30066607 PMC6675667

[B19] Ingram S. H. South S. C. (2020). Dimensional latent factor structure of psychopathology in adults with chronic illness. Psychol. Assess. 32, 1145–1157. 10.1037/pas0000954 33271041

[B20] Lenzo V. Sardella A. Martino G. Quattropani M. C. (2020). A systematic review of metacognitive beliefs in chronic medical conditions. Front. Psychol. 10:2875. 10.3389/fpsyg.2019.02875 31998178 PMC6965316

[B21] Mammarella I. C. Ghisi M. Bomba M. Bottesi G. Caviola S. Broggi F.. (2016). Anxiety and depression in children with nonverbal learning disabilities, reading disabilities, or typical development. J. Learn. Disabil. 49, 130–139. 10.1177/0022219414529336 24733818

[B22] Manti F. Giovannone F. Sogos C. (2019). Parental stress of preschool children with generalized anxiety or oppositional defiant disorder. Front. Pediatr. 7:415. 10.3389/fped.2019.00415 31681713 PMC6811652

[B23] Marchini F. Caputo A. Balonan J. T. Fedele F. Langher V. Napoli A. (2020). Emotional dynamics of persons with type 2 diabetes and their potential role in treatment adherence: insights from a clinical psychodynamic perspective. Psychol. Hub 37, 23–30. 10.1333/2724-2943/17160

[B24] Marchini F. Caputo A. Napoli A. Balonan J. T. Martino G. Nannini V.. (2018). Chronic illness as loss of good self: underlying mechanisms affecting diabetes adaptation. Mediterranean J. Clin. Psychol. 6. 10.6092/2282-1619/2018.6.1981

[B25] Martino G. Caputo A. Bellone F. Quattropani M. C. Vicario C. M. (2020a). Going beyond the visible in type 2 diabetes mellitus: defense mechanisms and their associations with depression and health-related quality of life. Front. Psychol. 11:267. 10.3389/fpsyg.2020.00267 32174865 PMC7054284

[B26] Martino G. Caputo A. Vicario C. M. Catalano A. Schwarz P. Quattropani M. C. (2020b). The relationship between alexithymia and type 2 diabetes: a systematic review. Front. Psychol. 11:2026. 10.3389/fpsyg.2020.02026 32982843 PMC7484475

[B27] Martino G. Catalano A. Agostino R. M. Bellone F. Morabito N. Lasco C. G.. (2020c). Quality of life and psychological functioning in postmenopausal women undergoing aromatase inhibitor treatment for early breast cancer. PLoS ONE 15:e0230681. 10.1371/journal.pone.0230681 32214378 PMC7098625

[B28] Martino G. Catalano A. Bellone F. Russo G. T. Vicario C. M. Lasco A.. (2019). As time goes by: anxiety negatively affects the perceived quality of life in patients with type 2 diabetes of long duration. Front. Psychol. 10:1779. 10.3389/fpsyg.2019.01779 31428028 PMC6689992

[B29] Mogentale C. Chiesa C. (2009). Esperienza di un trattamento combinato neuropsicologico sub-lessicale per la dislessia evolutiva [Experience of a combined sub-lexical neuropsychological treatment for developmental dyslexia]. Dislessia 6, 239–267.

[B30] Ryan M. (2006). Problemi sociali ed emotivi collegati alla dislessia [Social and emotional problems related to dyslexia]. Dislessia 3, 29–35.

[B31] Saklofske D. H. Austin E. J. Mastoras S. M. Beaton L. Osborne S. E. (2012). Relationships of personality, affect, emotional intelligence and coping with student stress and academic success: different patterns of association for stress and success. Learn. Individ. Differ. 22, 251–257. 10.1016/j.lindif.2011.02.010

[B32] Sorrenti L. Spadaro L. Mafodda A. V. Scopelliti G. Orecchio S. Filippello P. (2019). The predicting role of school Learned helplessness in internalizing and externalizing problems. An exploratory study in students with Specific Learning Disorder. Mediterranean J. Clin. Psychol. 7, 1–14. 10.6092/2282-1619/2019.7.2035

[B33] Steinmayr R. Crede J. McElvany N. Wirthwein L. (2016). Subjective well-being, test anxiety, academic achievement: testing for reciprocal effects. Front. Psychol. 6:1994. 10.3389/fpsyg.2015.01994 26779096 PMC4705295

[B34] Venkatesan S. (2017). Diagnostic decision tree for specific learning disability. Int. J. Educ. Psychol. Res. 6, 121–127.

[B35] Vicario C. M. Nitsche M. A. Salehinejad M. A. Avanzino L. Martino G. (2020). Time processing, interoception, and insula activation: a mini-review on clinical disorders. Front. Psychol. 11:1893. 10.3389/fpsyg.2020.01893 32973605 PMC7461974

[B36] Wang K. T. (2012). Personal and family perfectionism of Taiwanese college students: relationships with depression, self-esteem, achievement motivation, and academic grades. Int. J. Psychol. 47, 305–314. 10.1080/00207594.2011.626050 22150292

[B37] Wang Y. Wang G. Zhang N. Huang J. Wu W. Jia F.. (2020). Association between residual symptoms and social functioning in patients with depression. Compr. Psychiatry 98, 152–164. 10.1016/j.comppsych.2020.152164 32006810

[B38] Yoo J. Ryff C. D. (2019). Longitudinal profiles of psychological well-being and health: findings from Japan. Front. Psychol. 10:2746. 10.3389/fpsyg.2019.02746 31920803 PMC6914807

[B39] Zumbrunn S. Bruning R. (2013). Improving the writing and knowledge of emergent writers: the effects of self-regulated strategy development. Read. Writing 26, 91–110. 10.1007/s11145-012-9384-5

